# “Real-World” Practical Evaluation Strategies: A Review of Telehealth Evaluation

**DOI:** 10.2196/resprot.3459

**Published:** 2014-12-17

**Authors:** Stephen Agboola, Timothy M Hale, Caitlin Masters, Joseph Kvedar, Kamal Jethwani

**Affiliations:** ^1^Partners Healthcare Center for Connected HealthBoston, MAUnited States; ^2^Massachusetts General HospitalBoston, MAUnited States; ^3^Harvard Medical SchoolBoston, MAUnited States

**Keywords:** telehealth, eHealth, evaluation, evaluation framework, diabetes mellitus, technology

## Abstract

**Background:**

Currently, the increasing interest in telehealth and significant technological breakthroughs of the past decade create favorable conditions for the widespread adoption of telehealth services. Therefore, expectations are high that telehealth can help alleviate prevailing challenges in health care delivery. However, in order to translate current research to policy and facilitate adoption by patients and health care providers, there is need for compelling evidence of the effectiveness of telehealth interventions. Such evidence is gathered from rigorously designed research studies, which may not always be practical in many real-world settings.

**Objective:**

Our aim was to summarize current telehealth evaluation strategies and challenges and to outline practical approaches to conduct evaluation in real-world settings using one of our previously reported telehealth initiatives, the Diabetes Connect program, as a case study.

**Methods:**

We reviewed commonly used current evaluation frameworks and strategies, as well as best practices based on successful evaluative efforts to date to address commonly encountered challenges in telehealth evaluation. These challenges in telehealth evaluation and commonly used frameworks are described relevant to the evaluation of Diabetes Connect, a 12-month Web-based blood glucose monitoring program.

**Results:**

Designers of telehealth evaluation frameworks must give careful consideration to the elements of planning, implementation, and impact assessment of interventions. Evaluating performance at each of these phases is critical to the overall success of an intervention. Although impact assessment occurs at the end of a program, our review shows that it should begin at the point of problem definition. Critical to the success of an evaluative strategy is early planning that involves all stakeholders to identify the overall goals of the program and key measures of success at each phase of the program life cycle. This strategy should enable selection of an appropriate evaluation strategy and measures to aid in the ongoing development and implementation of telehealth and provide better evidence of program impact.

**Conclusions:**

We recommend a pragmatic, multi-method, multi-phase approach to telehealth evaluation that is flexible and can be adapted to the characteristics and challenges unique to each telehealth program.

## Introduction

### Background

Current global trends suggest that the popularity of telehealth is at an all-time high since the advent of modern telehealth over 40 years ago [1]. This rise is attributable to expectations that telehealth can help improve the current health care conundrum—rising prevalence of chronic diseases, shortage in health care workforce, and rising health care costs. These demands coincide with tremendous advancements in the technological landscape. Today, due to increasing affordability and user-friendliness of modern technologies, they have become ubiquitous. For example, more than 85% of the world’s population today has a mobile phone [2]. This abundance makes mobile phones a ready medium for health care delivery, capable of uniquely engaging patients to improve quality and access to care. Telehealth interventions come in various forms, ranging from simple one-way text messaging aimed at health education, to the collection and transmission of relevant biometric data used in home monitoring programs [3-5]. More recently, systems driven by complex algorithms using sensors and evidence-based psychological theories to motivate sustained behavior change are being developed, and early tests find evidence of better clinical and economic success [6].

Over the last decade, support has been growing for the use of telehealth. The ongoing health care reforms have significantly increased government funding as well as increased state legislation supporting expansion of telehealth services [7,8]. Consequently, adoption has also increased among insurers, health care providers, and professional associations. For example, home monitoring of blood pressure and blood glucose (BG) are now basic components of hypertension and diabetes management guidelines [9,10].

Despite the great strides made in the field, critics argue that evidence of the impact of telehealth is not strong [11]. The evidence of an intervention’s impact has been traditionally established by systematically and rigorously designed research studies. Randomized experiments that control for a variety of biases remain the gold standard for such evaluations. Policy makers often base their decisions on research from a meta-analysis of multiple evaluation efforts, where individual study estimates are pooled to estimate the overall effect of similar telehealth intervention programs. However, current telehealth literature is largely heterogeneous consisting of many pilots and mixed quality trials with diverse outcomes, which makes it difficult to estimate pooled effects of telehealth [12]. The time and resources required to test telehealth programs in randomized experiments is substantial, and with rapid changes in technology, impractical. In this paper, our goal was not to propose a new evaluation framework but rather to summarize current evaluation strategies in telehealth and outline practical steps to conducting evaluation in real-world settings using one of our previously reported telehealth initiatives, the Diabetes Connect program (DC), as a case study.

**Figure 1:**
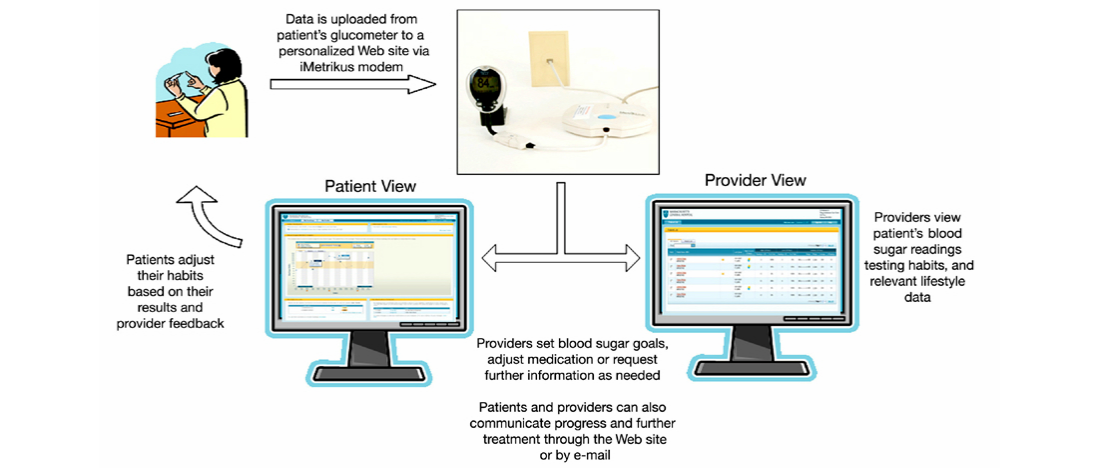
Diabetes Connect conceptual model.

Diabetes Connect is a 12-month Web-based blood glucose monitoring program. The program is designed to help improve blood glucose control by promoting patient self-management in the area of regular self-monitoring (a recommended diabetes self-care behavior) and facilitating patient-care provider connection (Figure 1). The program enables patients to easily collect and upload their blood glucose readings, via data transfer devices, to a secure Web-based platform. The real-time data sharing enables the care team to provide personalized and timely feedback to patients. Findings from this program are reported in previous publications [4,13,14]. The program was evaluated through all the stages of a telehealth project life cycle, and we found it helpful to follow the simple evaluative life cycle model described subsequently. Before describing the methods and results of the DC evaluation, we will briefly summarize some commonly used current evaluation frameworks and strategies.

### Overview of Current Evaluation Work, Challenges, and Emerging Frameworks

A number of program evaluation frameworks are available, depending on the phase of program development and the feasibility of data collection. In this paper, we provide a brief summary of challenges in telehealth evaluation and commonly used frameworks relevant to the evaluation of DC to orient investigators new to telehealth project design and program evaluation. For a comprehensive review of existing telehealth evaluation frameworks, see Ekeland et al [12] and van Gemert-Pijnen et al [15]. Bashshur et al describe four types: (1) evaluability assessment, (2) documentation evaluation, (3) formative or process evaluation, and (4) summative or outcome evaluation [16]. Each evaluation type is defined in Table 1 with an example based on DC.

**Table 1:** Evaluation typology.

Type	Definition	Key features and uses	Example
Evaluability	Assessment conducted prior to or at the beginning of a program to make explicit the goals and objectives of the program and intended effects or outcomes	Frame research question	Stakeholders (eg, investigators, health care professionals, case workers, and patients) meet to discuss goals of the program and identify key processes and outcomes that the program is intended to impact.
		Determine research design	
		Identify measurement tools and data collection methods	
		Determine analytic methods	
Documentation	A narrative description of the implementation of the program	Description of procedures and protocol used	The program is to be used at four clinics. Notes are taken on how the program is implemented across sites, barriers or difficulties to implementation, and any modifications to the overall program or site-specific adjustments.
		Description of difficulties encountered	
		Description of steps taken to address barriers to implementation	
		Identify successful strategies to dealing with barriers	
		Enable others to reproduce the program in other settings	
Formative or process	Evaluation focusing on the effects of the program on the process of care	Behavioral and attitudinal changes related to program adoption and use	A formative evaluation using interviews and focus groups with patients was conducted to examine if DC improved disease management, communication between patients and providers, and whether there were any unexpected barriers to effective use.
		Identify barriers to adoption and use	
		Resolve workflow integration issues	
		Identify technical problems	
Summative or outcome	Provides evidence of the intended effects of the program.	Robust evidence of program effects	The key objective of the DC program was to augment care with home monitoring of blood glucose. Summative evaluation was conducted to examine the effect of the intervention on clinical outcomes measured by HbA1c.
		Identify benefits of a program	
		Provide evidence to decision makers and policy makers of a program’s benefits	

To date, most telehealth research has been small-scale studies, and evaluation research has largely been formative and focused on process variables [16]. Although formative evaluations do not provide generalizable results on the impact of telehealth, they do provide useful information on the behavioral, attitudinal, and cognitive factors related to program implementation and subsequent outcomes [16].

There have been calls for larger, long-term telehealth studies that would provide measures amenable to summative evaluation examining the intended effect of a program. Perhaps the most important outcomes are health care costs, and the potential benefits and gains in efficiency that telehealth might bring to health care delivery processes [16]. Summative evaluations most often employ randomized controlled trial (RCT) designs and standardized measures to generate results that can be compared across studies.

There are many issues that complicate the ability to conduct telehealth program evaluations, particularly robust summative evaluations. We summarize these issues into three interrelated challenges: (1) the diversity of telehealth programs, (2) traditional research designs like RCTs are often impractical for telehealth evaluation, and (3) telehealth programs are complex and dynamic, and evaluation frameworks do not capture all process and outcome metrics satisfactorily.

The first challenge is that telehealth interventions are diverse and span a range of medical conditions, health care delivery problems, and types of intervention strategies, thereby making it difficult to specify the parameters of a standard telehealth program. This problem sometimes makes outcome metrics program specific and restricts the generalizability of findings. This is further complicated by the insufficient description of many telehealth interventions, which makes reproducibility and comparison of interventions very difficult [17]. The CONSORT-EHEALTH elaborates on this challenge and recommends a checklist to standardize reporting of component parts of telehealth interventions [17]. Also, technology is constantly changing and creates new opportunities for innovative programs. As a result, the field of telehealth is in “constant flux” [16] and is difficult to define. In addition, as telehealth is integrated more broadly as part of regular care delivery, it becomes more difficult to define telehealth as a distinct modality of care [16].

A second challenge is that an RCT, the most widely accepted evaluation methodology, is often impractical for telehealth programs [16,18]. The classic experimental RCT design features the randomization of subjects to intervention or control groups, carefully concealing or “blinding” subjects and investigators to intervention assignment, and pre- and post-intervention measurements. Evidence from RCTs provides the most robust method to establish cause and effect relationships and is less likely to be influenced by subjects or investigators. However, a number of issues limit the use of RCTs to evaluate telehealth. In many cases, the assignment to control or intervention cannot be concealed from subjects, health care professionals, or investigators. RCTs are also expensive and labor intensive to conduct, which often limits studies to small samples and short study durations [19]. Additionally, the rapid pace of changes in technology means that programs are sometimes obsolete by the time a long-term RCT has been completed. Other problems with how telehealth is implemented can introduce heterogeneity in the intervention and observed outcomes. For example, workflow and staffing across multiple sites is difficult to control and differences between sites can influence the observed effects [16].

The third challenge to telehealth evaluation is that health programs are more complex and dynamic than other types of interventions [16,20]. The evaluation of medication efficacy, for example, is a more clearly defined cause and effect relationship that is amenable to an experimental design. The success of complex health interventions like telehealth, however, is dependent on human behavior, program adoption, level of participant activation or engagement, and other contextual factors present where the intervention is implemented [20].

In response to these challenges, many current frameworks conceptualize telehealth as a complex health intervention that undergoes several stages of development, testing, and deployment. In addition, these frameworks highlight the role of stakeholders (eg, patients, caregivers, health care professionals and administrators, insurers, and state and federal agencies) and contextual factors in determining success. Khoja et al propose a framework that spans a telehealth program “life cycle” consisting of four phases: (1) development, (2) implementation, (3) integration, and (4) sustained operation [21]. Across the program life cycle, they identify several evaluation themes and associated outcomes. For example, during the implementation phase, one evaluation theme is “health services” with outcomes that include (1) improved diagnosis and treatment, (2) improved decision support, (3) better clinical safety, and (4) equity of care. Van Gemert-Pijnen et al propose a “holistic” framework that highlights the role of stakeholders as active participants in the development, implementation, and evaluation of telehealth programs [15]. The first step is a contextual inquiry to gather information from stakeholders to identify needs and gain insight for finding solutions. Findings from this step are further elaborated on by a value specification to identify the most favorable solutions. Through a process of continuous, iterative formative evaluations, a telehealth program can be tailored to fit the needs of users and the health care context. As the program matures, a summative evaluation is used to measure the success of the program based on the key goals and measures identified at the beginning of the project.

The RE-AIM Evaluation Framework (see Table 2) has been used to evaluate telehealth as well as other health programs [22-24]. It focuses on both the individual (Reach and Effectiveness) and organization level (Adoption, Implementation, and Maintenance) metrics to assess program impact. It translates research findings into action by encouraging stakeholders to focus on essential program elements and their external validity, not just the outcome of the research like many traditional models [22]. This enhances the quality, speed, and impact of the research in the real world, in turn, creating effective, generalizable, and evidenced-based interventions. Although the RE-AIM framework lacks a clear progression of program phases, it provides a flexible framework that identifies five elements common to health programs that determine success.

**Table 2:** RE-AIM elements and definitions.

RE-AIM element	Definition
Reach	The number and percent of people from the target population who participate, and their representativeness.
Effectiveness	The change in outcomes observed over the duration of the intervention.
Adoption	The number and percent of settings and staff who are expected to use the intervention and who participate.
Implementation	The extent to which the intervention is delivered consistently and the time and costs associated with implementation.
Maintenance	The long-term effects on key outcomes, and the extent to which a program is sustained, modified, or discontinued after the initial trial phase.

Rather than creating new methods for telehealth evaluation, many authors recommend greater standardization of existing methods and the use of multiple or mixed methods. Where possible, the standardization of study design, outcome measures, and analytic techniques would improve the ability to compare results between telehealth programs and conduct meta-analyses aimed at evaluating the field as a whole [12]. However, standardization of methods across the many types of telehealth programs has been difficult to achieve. To generate more robust conclusions of causality, the use of multiple methods, using both qualitative and quantitative research methods [25], and frameworks to “triangulate” the effects of telehealth programs have been suggested as a useful strategy [16].

In sum, these challenges highlight the need to carefully consider a practical set of goals and strategies for conducting telehealth evaluations in “real-world” settings, given the limitations of available resources and opportunities to collect data.

## Methods

### A Practical Approach to Conducting “Real-World” Telehealth Evaluation

**Figure 2:**
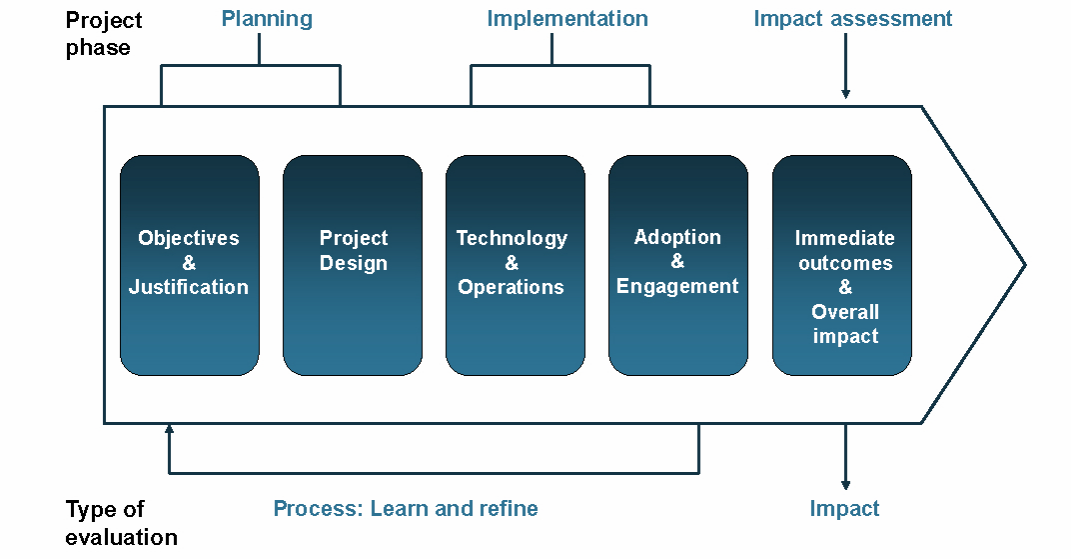
Telehealth program pathway.

We describe some best practices based on successful evaluative efforts to date to address commonly encountered challenges in telehealth evaluation: time, impracticality of RCTs, and the heterogeneity of implementation and diversity of outcomes across the program pathway. In its simplest form, the life cycle of any project involves three main phases with several activities: planning, implementation, and impact assessment (Figure 2). Evaluating performance at each of these phases is critical to the overall success of an intervention. Although impact assessment, the overall measure of effectiveness of the intervention, occurs at the end of a program, it should not be an afterthought but should begin at the point of problem definition. For this reason, fidelity to project protocol in implementing all three phases is a necessary prerequisite for a valid and methodologically sound evaluation [26].

### Planning

#### Objectives and Justification

The first step in planning is to define the goals and set the direction for the project using guidelines for SMART (Specific, Measurable, Achievable, Relevant, and Time-bound) goals and objectives [27]. Goals may be based on improving outcomes, increasing access, decreasing costs, or may simply be learning objectives and should guide the generation of performance indicators to evaluate the project. They are usually based on what may be of interest to funders and stakeholders, especially those who will accelerate the deployment of these programs at scale. At this stage, a consultation with a biostatistician may be necessary in specifying measurable endpoints. Our objectives were to determine the effect of the program on clinical outcomes (measured by changes in HbA1c, trends in BG measurements). Being one of our first experiences with remote monitoring for diabetes, we were also interested in general usability, satisfaction, and determinants of participant engagement in the program.

#### Project Design

Based on the outcomes expected, processes and activities required for project implementation should be defined. This includes identification of the target population, identification of stakeholders and role definition, design of project protocol, budgeting, staff allocations, setting timelines, etc. One must keep in mind that performance indicators should be generated for each step in the process.

After relevant outcome measures are agreed upon, a suitable evaluation design should be identified. Some goals, like engagement or adoption metrics, can be interpreted without any comparison data. However, for most other goals, including access and clinical outcomes, a comparison arm is required. Randomized control groups are considered to be the gold standard, but a variety of other methodologies have been used in telehealth evaluation, albeit with certain limitations. Paired analyses before and after the intervention, matched control analysis, as well as using existing population-level metrics for comparison have all been used with varying success [28]. These options should be considered when faced with time or monetary constraints in using an RCT approach.

### Implementation

This is usually the longest phase in a project and consists of assigning inputs and generating outputs for project execution.

#### Technology and Operations

Project objectives and design will inform the decisions that need to be made about technology and operational processes, which are the main inputs needed to perform project activities. Inputs, by definition, are the resources needed to support the primary activities of the project, and they include funding, staff and their expertise, technology, and other materials needed for project implementation. During this time, careful consideration must be given to select the “right” technology to develop an effective telehealth intervention [18] and also to ensure that all operational processes conform to the pre-specified plan. All protocol deviations should be documented and reported to the principal investigator and appropriate institutional review board staff in a timely fashion. Furthermore, the technology should be evaluated for its ability to function as specified and integrate adequately with other technologies and existing systems, as well as it timely availability, usability, and acceptability. In addition, it is also helpful to be vigilant to disruptions in workflow processes or system malfunction. These and other technical problems reported by users should be logged, reported, and addressed promptly.

Once the technology has been chosen, deployment strategies can be designed. Program adoption and engagement can vary widely based on who offers the technology to patients, how it is set up, and the availability of customer service after initial deployment. Measures for each of these assumptions and requirements should be made part of the evaluation plan.

#### Adoption and Engagement

Strategies relating to major stakeholder (patient and provider) engagement and adoption are critical elements of a telehealth intervention. Rogers describes an “innovation-decision” process involved in adopting or rejecting a new intervention [29] and defines adoption as the decision of stakeholders to make full use of an innovation as the best course of action available [29]. Subsequently, stakeholders begin to engage with the intervention. Engagement refers to “actions individuals must take to obtain the greatest benefit from the health care services available to them” [30]. Evidence exists that provider and patient engagement are interlinked, and thought must be given to measuring both appropriately. For patients, themes to include are literacy, intrusiveness, user interface design, simplicity, and usability of intervention. For providers, integration with existing workflow, appropriateness of personnel delegation, electronic medical record integration, and ease of program deployment should be considered. Also, behavioral themes like activation and readiness to change are critical factors in optimizing engagement. Examples of techniques to improve user engagement include personalization, behavioral economics (like rewards/incentives, nudges), gamification, and social networks.

### Impact Assessment

This phase includes measurement of both intermediate outcomes and overall effects (impact assessment) of the intervention. Intermediate outcomes are direct effects attributable to project outputs, like increased knowledge, increased adherence to recommended treatments, behavior change, early detection, and treatment of symptoms. Often they may be used as proxies to measure endpoints. In cases where objective measurements of effects may be difficult to assess quantitatively, common qualitative approaches, like patient interviews or focus groups, can be used to more fully measure the effects attributable to the intervention and are particularly useful for assessing subjective patient preferences.

Impact assessment, on the other hand, estimates the net impact and overall effect of the intervention. They may be clinical or financial and are usually more meaningful to stakeholders. As a rule of thumb, the more rigorous the research methodology, the more accurate the effect estimate. However, in practice, the most rigorous design is not usually feasible. The goal should be to achieve a balance between managing real-world constraints and the next best possible design. Therefore, based on the setting and other limiting factors where the telehealth intervention is deployed, alternative study designs, which maximize efficiency, validity, and reproducibility while minimizing risk of bias should be considered (Textbox 1).

**Strategies to minimize risk of bias.**

- considering multi-site trials to address the problem of small sample sizes [18]
- using validated questionnaires/tools to assess impact [18]
- using unobtrusive data collection techniques or routine clinical data [18]
- using both quantitative and qualitative approaches to completely and accurately estimate effects of intervention [12]
- considering formative approaches to improve next iteration of the project to increase scalability potential and maintain ongoing success
- using alternative research designs including quasi-experimental designs, matched control trials, staged interventional trials, crossover trials, etc [28]

## Results

### Planning: Objectives and Justification

In our DC program, we were interested in whether augmenting regular care with home monitoring of BG (thereby enabling patient self-management and providing clinicians with home BG data in between office visits) improved clinical outcomes as measured by change in HbA1c. This goal definition further helped us to specify program objectives and outcome variables. Our objectives were to determine the effect of the program on clinical outcomes (measured by changes in HbA1c, trends in BG measurements). Being one of our first experiences with remote monitoring for diabetes, we were also interested in general usability, satisfaction, and determinants of participant engagement in the program.

### Implementation

#### Technology and Operations

In DC, the adopted technologies integrated well with existing systems and data transfer was confirmed within 24 hours of set-up. Technical personnel were available during business hours to answer questions or help troubleshoot for ongoing problems. Tracking the frequency of troubleshooting calls was particularly important for us to identify some differences early in the study in the pattern of participants’ interactions with the technologies. We also found it very important for staff to standardize all practices, time log all problems, and track them until resolution. These processes were important for continuous system improvement.

A trial of the initial version of the DC in a pilot phase before mass enrollment of participants helped us identify early any problem areas in deployment, adoption, and engagement that required further work.

#### Adoption and Engagement

In this phase, some of the key evaluation questions in DC included:

- Was the target audience appropriately reached?

- Has the program been adopted by key stakeholders?
- Are key stakeholders engaged?
- Are engagement patterns different based on time, demographic group, or location?
- Are some participants responding differently to engagement techniques than others?
- Are ongoing troubleshooting issues reported and addressed promptly?

DC was adopted by clinicians in four different practices within the Partners HealthCare network of hospitals who enrolled suitable patients in the program for 12 months. Help desk support was available (Monday to Friday, business hours) to address in a timely fashion any technical issues that might arise. Patient engagement was assessed by number of uploads and BG readings recorded by the DC. We also assessed provider and practice engagement by assessing the number of times providers logged on to the Web portal to view patients’ BG readings and the average number of provider logins to the Web portal by practice.

### Impact Assessment

We used focused groups as a measure with DC because they allowed us to capture contextual content on participants’ experiences, understandings, perspectives, or stories we might have otherwise missed in personal interviews. They facilitated lively conversations around usage, interactions with clinicians, and difficulties they encountered as they interacted with the system. We found focus groups particularly helpful to evaluate program perception, engagement, and adoption. From these groups, we discovered that most participants’ favorite part of the program was the ability to easily send BG readings to their providers and when appropriate, they appreciated the timely feedback. Additionally, participants reported that their provider’s interest in the program was the strongest predictor of whether or not they would use the program. These findings would have been nearly impossible to discover without the focus group and were very helpful in future program development.

A critical assumption in impact assessment is the availability of high-quality data. We ensured that our endpoint data were collected appropriately and in a timely manner. Data points from DC include HbA1c, BG readings, frequency of data uploads, and frequency of logins to the portal. These data were vital for continued program improvement and outcome evaluation. For example, we found that the mean changes in HbA1c showed promising results: those who uploaded more BG readings had a further decline in their HbA1c. Additionally, the nature of DC made measuring engagement quantitatively through more than one metric simple, and we successfully measured the frequency of uploads by participants as well as logins by both participants and providers. We also used qualitative methods to assess our outcomes in participants recruited from four different sites. Through the focus groups, users and potential users of the program were interviewed to obtain feedback including comfort level using the system and how the program could be better tailored to their needs. Knowing what information was most important to collect during the program-planning phase made the impact assessment process easier to conduct, and we were able to detect meaningful changes in our outcome measures.

## Discussion

### Principal Considerations

We have provided a brief review of evaluation strategies, drawing on our experiences in creating and evaluating telehealth programs to describe a practical approach to conducting evaluations in real-world settings. We recommend that each telehealth program have an evaluation procedure in place at every stage of the program life cycle, from ideation to large-scale deployment and impact assessment, while considering the different characteristics and challenges unique to each telehealth program. Key to implementing this strategy is early planning involving all stakeholders to define overall goals and goals for each project phase. Once consensus is reached, an evaluation design should be selected that best fits the identified goals and the constraints of project resources. Early planning is essential to ensure valid measurement of goals across program phases and to evaluate overall impact.

The telehealth evaluation strategy we described has two distinct advantages over classic, large-scale RCT studies. First, telehealth interventions are complex health interventions that can be best understood as “works in progress” tailored to fit the unique needs of each site and set of users. Telehealth evaluation across the program life cycle can provide useful data to guide program design and improve the reach, effectiveness, and adoption of new interventions. This evaluation strategy may accelerate the translation of research into practice to create programs that are well integrated into existing clinical workflows to improve efficiency and ultimately control health care costs. The results from formative/process evaluations are often key to developing telehealth programs with high levels of sustained user adoption and engagement. Patient engagement is an important objective in creating telehealth programs that focus on lifestyle change, improved patient self-management, and the prevention and control of chronic disease.

Second, greater participation by stakeholders early in the program planning process may foster sustained involvement and commitment to telehealth programs during implementation and large-scale deployment. This could increase the number of telehealth programs that are successfully transitioned to larger programs integrated into standards of care practices.

Some of the lessons we learned from DC and our other telehealth programs that have proven to be useful over the years are found in Textbox 2.

**Keypoints from Diabetes Connect and other telehealth programs.**

- Start thinking about evaluation early and involve as many stakeholders as possible, including participants, funders, etc.
- Design your project based on proposed outcomes. Keep in mind the requirements to collect adequate data at the appropriate times and the need for objective metrics to evaluate each process in the program.
- Consider the limitations of the evaluation design. An RCT may not always be feasible, so explore other program design options, keeping in mind resource considerations and internal validity.
- Technology is essential, but not the focus of the program. Evaluate whether you are using the right technology based on ease and appropriateness of use, integration capabilities, and scalability. Remember that the final goal is to use something that your target population will adopt easily and engage with in the long term.
- Engagement in the program is key. Remember to include proven elements in your program design (like personalization, behavioral economics, etc) as well as metrics to evaluate their effectiveness in your evaluation plan.
- To maximize the potential of a valid, reliable evaluation, choose program design elements that reduce the potential for bias, like using validated survey instruments, using both qualitative and quantitative assessment methodologies, etc.

### Conclusions

Efforts to evaluate telehealth programs create what Bashshur et al describe as a “quandary” [16]. On one hand, RCTs are the consensus method for conducting rigorous evaluation research. However, they are always not well suited or practical for telehealth evaluation [16,25,31]. We recommend a pragmatic, multi-method, multi-phase approach that is flexible and can be adapted to the characteristics and challenges unique to each telehealth program as showcased in the Diabetes Connect program. Critical to the success of this strategy is early planning that involves all stakeholders to identify the overall goals of the program and key measures of success at each phase of the program life cycle. Following this strategy should enable investigators to select an appropriate evaluation strategy and measures that will aid in the ongoing development and implementation of telehealth and provide better evidence of program impact.

## Abbreviations

BG: blood glucose
DC: Diabetes Connect
RCT: randomized controlled trial
